# What Profession Shall a Young Man Choose?

**Published:** 1881-10

**Authors:** 


What Profession Shall a Young Man Choose?—We call the attention of our readers to the exceedingly able address of Professor Bates, printed in another part of this Journal. It contains the first complete and authentic history of veterinary education in this country that has ever been written. More than that, it presents in a most effective way the facts regarding the needs and the purposes of American veterinary medicine.

It is not necessary to repeat here the statements made and the statistics given by Dr. Bates. They all show the necessity to our country of more veterinarians, and the sure promise of success to those who faithfully undertake the practice of comparative medicine.

We are pleased to have received from our esteemed correspondent, Dr. John Navin, some words upon this subject. Dr. Navin's ripe experience and sound judgment well qualify him to say what veterinary medicine needs and what it promises to those who educate themselves in it. The veterinary profession is the only one that is not overcrowded, while paying well in money, dignity, and honor. These are the sentiments of our Western Nestor, and they are what every one who is acquainted with the facts must echo.

